# Obicetrapib: Reversing the Tide of CETP Inhibitor Disappointments

**DOI:** 10.1007/s11883-023-01184-1

**Published:** 2023-12-22

**Authors:** John J. P. Kastelein, Andrew Hsieh, Mary R. Dicklin, Marc Ditmarsch, Michael H. Davidson

**Affiliations:** 1 NewAmsterdam Pharma B.V., Naarden, Netherlands; 2 Midwest Biomedical Research, Addison, IL USA

**Keywords:** Cholesteryl ester transfer protein (CETP) inhibitor, Obicetrapib, Atherosclerotic cardiovascular disease (ASCVD), Low-density lipoprotein cholesterol (LDL-C), High-density lipoprotein cholesterol (HDL-C), Cardiovascular outcomes trial

## Abstract

**Purpose of Review:**

To discuss the history of cardiovascular outcomes trials of cholesteryl ester transfer protein (CETP) inhibitors and to describe obicetrapib, a next-generation, oral, once-daily, low-dose CETP inhibitor in late-stage development for dyslipidemia and atherosclerotic cardiovascular disease (ASCVD).

**Recent Findings:**

Phase 1 and 2 trials have evaluated the safety and lipid/lipoprotein effects of obicetrapib as monotherapy, in conjunction with statins, on top of high-intensity statins (HIS), and with ezetimibe on top of HIS. In ROSE2, 10 mg obicetrapib monotherapy and combined with 10 mg ezetimibe, each on top of HIS, significantly reduced low-density lipoprotein cholesterol (LDL-C), non-high-density lipoprotein cholesterol (non-HDL-C), apolipoprotein B, total LDL particles, small LDL particles, small, dense LDL-C, and lipoprotein (a), and increased HDL-C. Phase 3 pivotal registration trials including a cardiovascular outcomes trial are underway.

**Summary:**

Obicetrapib has an excellent safety and tolerability profile and robustly lowers atherogenic lipoproteins and raises HDL-C. As such, obicetrapib may be a promising agent for the treatment of ASCVD.

## Introduction

Cholesterol in the plasma circulates predominantly as cholesteryl esters. Humans, non-human primates, and a few other animal species possess cholesteryl ester transfer protein (CETP), a plasma glycoprotein secreted by the liver that mediates the bidirectional transfer of cholesteryl esters and triglycerides between high-density lipoprotein (HDL), very low-density lipoprotein (VLDL), and low-density lipoprotein (LDL) particles. CETP activity tends to result in a net mass transfer of cholesteryl esters from HDL to VLDL and LDL, and a net mass transfer of triglycerides from VLDL to LDL and HDL [1]. Inhibiting CETP reduces these exchanges resulting in increased concentrations of cholesterol in HDL and decreased concentrations of cholesterol in apolipoprotein (Apo) B-containing particles, i.e., VLDL and LDL [1].

Genetic deficiency of CETP activity, and its association with an improved lipid profile (decreased LDL cholesterol (LDL-C) and increased HDL cholesterol [HDL-C]) resulting in reduced premature atherosclerosis, was first described in Japanese subjects in the mid-1980s [2–5]. This led to interest in the development of pharmacologic CETP inhibitors. Epidemiological studies have shown that LDL-C is directly and HDL-C is inversely associated with the risk of atherosclerotic cardiovascular disease (ASCVD) [6–10]. In contrast to LDL, HDL is known to have important roles in reverse cholesterol transport, which is the removal of cholesterol from peripheral tissues to the liver, and to have anti-inflammatory, anti-thrombotic, anti-oxidative, and anti-atherogenic properties [11, 12]. Nevertheless, results from randomized controlled trials of therapies that predominantly raise HDL-C, such as niacin and fibrates, have not supported the HDL-C hypothesis for reduced risk of ASCVD, particularly as add-on therapy to statins in today’s medical environment [13–21].

Cardiovascular outcomes trials of early CETP inhibitors, including torcetrapib, dalcetrapib, and evacetrapib, were unsuccessful in demonstrating reduced risk of ASCVD for a variety of agent-specific, not class-specific, reasons [1, 22–27]. However, the Randomized Evaluation of the Effects of Anacetrapib Through Lipid Modification (REVEAL) trial showed a significantly lower incidence of major coronary events with anacetrapib vs. placebo among patients with ASCVD who were receiving intensive statin therapy [28]. This result was tied to its effect on ApoB particles, rather than HDL-C [28, 29•, 30]. The developer of anacetrapib concluded that the “clinical profile for anacetrapib does not support regulatory filings” and did not apply for regulatory approval [31] due to anacetrapib’s accumulation in the adipose tissue because of its high lipophilicity [32]. Other CETP inhibitors in development since the discontinuation of anacetrapib include CKD-508, MK-8262, and obicetrapib (TA-8995), which has reached phase 3 clinical development [1, 33, 34, 35•, 36–38].

Obicetrapib, a next-generation, oral, once-daily, low-dose CETP inhibitor under development for the treatment of dyslipidemia, cardiovascular risk, and Alzheimer’s disease, is reversing the tide of largely negative findings for CETP inhibition and is on the path towards being the first-in-class CETP inhibitor available for clinical use. It potently lowers LDL-C, non-HDL-C, ApoB, LDL particle concentration, particularly small LDL particles, and lipoprotein(a) (Lp(a)) and raises pre-beta HDL as well as mature HDL particles and ApoA-1 and ApoE [29•, 35•, 39–41, 42•]. These effects are evident not only with obicetrapib monotherapy but also in conjunction with medium dose statins, on top of high-intensity statins, and in combination with ezetimibe on top of high-intensity statins. This article provides an overview of the history of CETP inhibitors for reducing ASCVD risk and describes the promising development program of obicetrapib, including results from phase 1 and phase 2 trials, the objectives of ongoing phase 3 trials, and the known and hypothesized mechanisms of action for obicetrapib in combination with statins and ezetimibe.

## History of the Development of CETP Inhibitors for ASCVD Risk Reduction

To date, five CETP inhibitors including torcetrapib, dalcetrapib, evacetrapib, anacetrapib, and, most recently, obicetrapib have reached late-stage clinical development for ASCVD risk reduction. The first CETP inhibitor to be assessed in phase 3 clinical trials, torcetrapib, increased the risks of cardiovascular events and death in the Investigation of Lipid Level Management to Understand its Impact in Atherosclerotic Events (ILLUMINATE) trial, which led to the trial’s premature termination and discontinuation of the development of torcetrapib [22]. At the time ILLUMINATE was terminated, the mechanism of increased risk was unknown. However, later it was determined that torcetrapib had structure-related off-target effects causing increased blood pressure, as well as increased aldosterone, steroid, and endothelin-1 levels, and electrolyte abnormalities [43]. None of the CETP inhibitors developed after torcetrapib has had the features that caused torcetrapib’s off-target effects, and all have demonstrated favorable safety profiles [23, 25, 28, 35•, 39, 42•, 44].

Cardiovascular outcomes of the next CETP inhibitor, dalcetrapib, were evaluated in the Randomized, Double-blind, Placebo-controlled Study Assessing the Effect of RO4607381 on Cardiovascular Mortality and Morbidity in Clinically Stable Patients with a Recent Acute Coronary Syndrome (Dal-OUTCOMES) [23]. A pre-specified interim analysis, after reaching 71% of the projected total number of events, led to the decision to terminate the trial for futility based on its failure to significantly affect any component of the primary endpoint (composite of death from coronary heart disease, nonfatal myocardial infarction, ischemic stroke, unstable angina, or cardiac arrest with resuscitation) or total mortality. HDL-C increased from baseline by 31–40% with dalcetrapib compared with a 4–11% increase in the placebo group, but dalcetrapib had minimal or no effects on LDL-C and ApoB.

The next CETP inhibitor to reach late-phase clinical development, evacetrapib, was evaluated in the Assessment of Clinical Effects of Cholesteryl Ester Transfer Protein Inhibition with Evacetrapib in Patients at a High-Risk for Vascular Outcomes (ACCELERATE) [25]. Like Dal-OUTCOMES, ACCELERATE was terminated early after 82% of the planned primary endpoint events (composite of death from cardiovascular causes, myocardial infarction, stroke, coronary revascularization, or hospitalization for unstable angina) because of lack of efficacy. However, compared to dalcetrapib, evacetrapib produced a larger increase in HDL-C (133% for evacetrapib vs. + 1.6% for placebo) and larger decreases in LDL-C (– 31.1% for evacetrapib vs. + 6.0% for placebo; direct LDL assay) and ApoB (– 15.5% for evacetrapib vs. + 3.8% for placebo). The LDL-C reduction was severely overestimated and taking the 15% reduction of ApoB into account, the LDL-C reduction was in fact between 15 and 18%. Evacetrapib also reduced Lp(a) (– 22.3% with evacetrapib vs. 0% for placebo). The reasons suggested for evacetrapib’s failure to reduce cardiovascular outcomes was the trial’s relatively short duration in conjunction with a very modest effect on LDL-C. The trial was ended after a mean of 26 months, with a mean absolute ApoB reduction of 12.1 mg/dL, which was almost identical to the absolute ApoB reduction in the Improved Reduction of Outcomes: Vytorin Efficacy International Trial (IMPROVE-IT) [45]. IMPROVE-IT had a median follow-up period of 7 years, and there was no separation in the Kaplan–Meier curves for cardiovascular events between the simvastatin monotherapy and simvastatin + ezetimibe arms until approximately 3 years into the treatment period [45].

The most recently completed cardiovascular outcomes trial of a CETP inhibitor, the Heart Protection Study (HPS)3/Thrombolysis in Myocardial Infarction (TIMI)55–Evaluation of the Effects of Anacetrapib through Lipid Modification (REVEAL) trial, demonstrated that adding anacetrapib to intensive atorvastatin therapy for a median follow-up period of 4.1 years significantly reduced the primary endpoint of a first major coronary event (composite of coronary death, myocardial infarction, or coronary revascularization) by 9% compared to adding placebo [28]. An extension of the follow-up period for an additional 2.2 years (median) demonstrated a further 20% reduction in coronary events [44]. Due to its accumulation in adipose tissue, anacetrapib has a long terminal half-life, which enabled the blinded, randomized treatment assignments in REVEAL to be maintained throughout the extended follow-up period [46, 47]. The combined overall proportional reduction in major coronary events over the full 6.3 years median follow-up was 12% [44]. In a subgroup analysis of the REVEAL results examining the effects of anacetrapib on major coronary events according to tertiles of baseline non-HDL-C, subjects receiving anacetrapib in the highest non-HDL-C tertile (≥ 101 mg/dL) had a much higher reduction in coronary events, 16%, compared to the overall cohort in which the mean baseline non-HDL-C level was 92 mg/dL and subjects receiving anacetrapib experienced a 9% reduction in events [28].

In REVEAL, LDL-C was decreased by 17% (beta-quantification), non-HDL-C by 18%, ApoB by 18%, and Lp(a) by 25%, and HDL-C was increased by 104% [28]. The difference in LDL-C between the anacetrapib and placebo arms of 11 mg/dL and the primary endpoint in REVEAL fully align with the Cholesterol Treatment Trialists’ meta-regression line for predicting the relationship between non-HDL-C and major adverse cardiovascular events based on results of statin trials [28, 48].

Although the initial focus during the development of CETP inhibitors for reducing risk of ASCVD was on their HDL-C-raising effects, the focus is now firmly placed on their ability to lower LDL-C, non-HDL-C, and ApoB, as supported by comprehensive evidence from animal models, observational cohorts, Mendelian randomization studies, randomized controlled trials, and large meta-analyses [29•, 49, 50]. The newest CETP inhibitor to reach late-phase clinical development, obicetrapib, has been shown to robustly reduce LDL-C, non-HDL-C, ApoB, and Lp(a) levels, as well as increase HDL-C, pre-beta and mature HDL particles, ApoA1, and ApoE [35•, 39, 40, 42•]. An overview of the clinical development program for obicetrapib follows.

## Development of Obicetrapib

Obicetrapib is a tetrahydroquinoline derivative with a pyrimidine and ethoxycarbonyl structure with two chiral centers. When compared to anacetrapib and evacetrapib at equipotent dosages, and when comparing 10 mg obicetrapib with 100 mg anacetrapib, obicetrapib has been shown to reduce CETP activity to a greater extent [39, 51–53]. This higher potency might be explained by obicetrapib’s less lipophilic structure [54]. Crystallography experiments show that CETP inhibitors generally locate at the N-terminal neck of the hydrophobic tunnel of CETP and can restrict the lipid flow through this tunnel [54]; however, because obicetrapib has a more polar structure, it may also interact with polar residues at the center of the inhibitor-binding site thereby improving its binding, specificity, and solubility [42•, 54].

Results from non-clinical pharmacology, pharmacokinetic, and safety studies supported the progression of obicetrapib into clinical development, which, to date, has included nine phase 1 studies in healthy volunteers (NewAmsterdam Pharma data on file for TA-8995–09, TA-8995–10, and OBEZ-101) [39, 55–58]; six completed phase 2 trials in patients with dyslipidemia or elevated lipoprotein(a) [35•, 40, 42•, 59–62], and three ongoing phase 3 trials [36–38]. Another therapeutic area for which obicetrapib is being investigated is Alzheimer’s disease; a phase 2a, proof-of-concept, open-label study in patients with early Alzheimer’s disease is ongoing [63].

### Phase 1 Trials of Obicetrapib

In the first-in-human, single ascending dose study (in Caucasian and Japanese volunteers) and a multiple ascending dose study of obicetrapib (Caucasians), which investigated single doses up to 150 mg and repeat doses up to 25 mg, respectively, no clinically significant effects of obicetrapib on vital signs, blood pressure, or biochemistry assessments, including aldosterone, sodium, potassium, or bicarbonate concentrations, which were safety concerns with torcetrapib, were observed [22, 39]. Furthermore, after 2.5 to 25 mg once-daily dosing, obicetrapib resulted in nearly complete inhibition of CETP activity (92–99%), increased HDL-C by 96–140%, and decreased LDL-C by 40–53%. There were no significant effects of age, gender, ethnicity, or food on the pharmacokinetics or pharmacodynamics of obicetrapib in these studies. A summary of the LDL-C, non-HDL-C, and ApoB responses with obicetrapib monotherapy in the multiple-ascending dose study and in other selected phase 1/2 trials is shown in Table 1.

**Table 1:** Median percent changes from baseline in lipoprotein lipid biomarkers for obicetrapib 10 mg monotherapy across phase 1 and 2 studies [35•, 39, 40, 42•, 62] (TA-8995–06, NewAmsterdam Pharma, data on file)

Trial	*N* per arm	Median % changes from baseline		
		LDL-C	Apo B	Non-HDL-C
Phase 1 MAD	10	− 44.1	− 29.6	−
TULIP	35	− 46.4	− 35.8	− 41.7
TA-8995–06	13	− 42.9	− 26.3	− 32.7
ROSE	40	− 50.8	− 29.8	− 44.4
ROSE2	26	− 43.5	− 24.2	− 37.5
Japan	26	− 45.8	− 29.7	− 37.0

*Abbreviations*: *ApoB*, apolipoprotein B; *LDL-C*, low-density lipoprotein cholesterol; *MAD*, multiple ascending dose; *N*, number of subjects; *Non-HDL-C*, non-high-density lipoprotein cholesterol;* ROSE*, Randomized Study of Obicetrapib as an Adjunct to Statin Therapy; *ROSE2*, Study to Evaluate the Effect of Obicetrapib in Combination with Ezetimibe as an Adjunct to High-Intensity Statin Therapy; *TA-8995-06*, A Study on the Effects of TA-8995 on Lp(a) in Subjects with Elevated Lp(a); *TULIP*, TA-8995: Its Use in Patients with Mild Dyslipidaemia

Additional phase 1 studies of obicetrapib included a thorough QT study which showed it had no effect on the 12-lead electrocardiogram heart rate-corrected QT interval using Fridericia’s formula [56]; a drug-drug interaction study that showed it is a mild inducer of cytochrome P450 3A4, but has no significant effect on P-glycoprotein activity [57]; and a mass balance recovery, pharmacokinetics, metabolism, and excretion study that concluded it is steadily absorbed and its principal route of excretion is in the feces [58] (NewAmsterdam Pharma, data on file). When the dosing formulation of obicetrapib was changed from a capsule to a tablet, the bioequivalence of 5 mg of each was confirmed [55] (NewAmsterdam Pharma, data on file). An investigation of the effects of food on the bioavailability of 10-mg obicetrapib tablets, was also conducted, which demonstrated 55–59% greater exposure to obicetrapib under fed (high-fat, high-calorie breakfast) vs. fasted conditions, suggesting that obicetrapib may be dosed with or without food (TA-8995–09, NewAmsterdam Pharma, data on file). Most recently, a single- and multiple-dose study was conducted on Chinese subjects; results demonstrated that obicetrapib was well tolerated and that its primary pharmacokinetic and pharmacodynamic parameters were similar to those in Caucasian subjects (TA-8995–10, NewAmsterdam Pharma, data on file). Finally, a pilot study of two fixed-dose combination formulations of obicetrapib 10 mg/ezetimibe 10 mg has been conducted, which demonstrated bioequivalence for the area under the curve from zero to infinity for both obicetrapib and ezetimibe in one of the formulations, but the values for maximum concentration failed to meet the acceptance criteria (OBEZ-101, NewAmsterdam Pharma, data on file). Additional phase 1 trials to further characterize the pharmacodynamics of obicetrapib are also planned, including the Investigating the Effect of Obicetrapib on Lipoprotein Metabolism study which aims to determine the effect of 10 mg obicetrapib added to background statin therapy on the fractional catabolic rate of ApoB in LDL among subjects with normal lipids or dyslipidemia on stable atorvastatin or rosuvastatin [64].

### Phase 2 Trials of Obicetrapib

The first phase 2 trial of obicetrapib, TA-8995: Its Use in Patients with Mild Dyslipidaemia (TULIP) conducted in Denmark and the Netherlands, administered daily obicetrapib monotherapy doses of 1, 2.5, 5, or 10 mg, and obicetrapib 10 mg in combination with 20 mg atorvastatin or 10 mg rosuvastatin, compared with placebo or statin alone, for 12 weeks to patients with mild dyslipidemia [40, 41]. Both the 5-mg and 10-mg obicetrapib monotherapy doses resulted in a significant 45% median reduction from baseline LDL-C, and with the 10-mg dose, Apo B was significantly reduced by 34% and Lp(a) by 33%, and HDL-C was increased by 179%. Because TULIP and previous studies in healthy subjects had demonstrated significant reductions in Lp(a) levels with obicetrapib, a dedicated investigation of the effects of obicetrapib 2.5 mg and 10 mg administered for 12 weeks, vs. placebo, on Lp(a) in subjects with elevated Lp(a) levels was conducted [59]. While both doses significantly reduced Lp(a) compared with placebo, the effects were smaller in magnitude than those seen previously (TA-8995–06, NewAmsterdam Pharma, data on file). Another dose-finding study of obicetrapib was conducted in Japanese subjects where obicetrapib 2.5, 5, and 10 mg was administered for 8 weeks as an adjunct to stable statin therapy (atorvastatin 10 or 20 mg or rosuvastatin 5 or 10 mg) [60]. At the 10-mg dose, median LDL-C was decreased by 46% and ApoB by 30%, and HDL-C was increased by 159% [62].

The combination of obicetrapib with high-intensity statins and ezetimibe has been evaluated in three phase 2 trials of patients with dyslipidemia: obicetrapib on top of high-intensity statin (Randomized Study of Obicetrapib as an Adjunct to Statin Therapy (ROSE)) [42•], obicetrapib in combination with ezetimibe (Randomized Study of Obicetrapib in Combination with Ezetimibe (OCEAN)) [61], and obicetrapib in combination with ezetimibe on top of high-intensity statins (Study to Evaluate the Effect of Obicetrapib in Combination with Ezetimibe as an Adjunct to High-Intensity Statin Therapy (ROSE2)) [35•]. Results from ROSE which administered 5 and 10 mg obicetrapib for 8 weeks in combination with high-intensity statins demonstrated that median LDL-C was significantly decreased by up to 51%, ApoB by up to 30%, and non-HDL-C by up to 44%, and HDL-C was significantly increased by up to 165% [42•]. Results from OCEAN demonstrated 34% and 52% LDL-C reductions with 5 mg obicetrapib alone and in combination with 10 mg ezetimibe, respectively, after an 8-week treatment period (TA-8995–303, NewAmsterdam Pharma, data on file). Results from ROSE2, which examined 10 mg obicetrapib alone and in combination with 10 mg ezetimibe, respectively, taken for 12 weeks on top of high-intensity statins demonstrated significant reductions in LDL-C, non-HDL-C, ApoB, total and small LDL particles, and Lp(a), as shown in Table 2, and significantly increased HDL-C by up to 142% [35•]. LDL-C levels of < 100, < 70, and < 55 mg/dL were achieved by 100%, 93.5%, and 87.1%, respectively, of patients taking the combination of obicetrapib plus ezetimibe on top of high-intensity statin [35•]. This supports the potential for obicetrapib to fill the treatment gap for patients with elevated LDL-C who are unable to achieve treatment objectives with currently available therapies [65–67].

**Table 2:** Median percent changes from baseline in atherogenic lipoprotein levels in the Study to Evaluate the Effect of Obicetrapib in Combination with Ezetimibe as an Adjunct to High-Intensity Statin Therapy (ROSE2) [35•] (TA-8995–202, NewAmsterdam Pharma, data on file)

	Median % changes from baseline		
	Placebo (*N* = 40)	Obicetrapib 10 mg (*N* = 26)	Obicetrapib 10 mg + ezetimibe 10 mg (*N* = 31)
LDL-C	− 6.35	− 43.5	− 63.4
Non-HDL-C	− 5.55	− 37.5	− 55.6
ApoB	− 2.05	− 24.2	− 34.4
Total LDL-P	− 5.65	− 54.8	− 72.1
Small LDL-P	− 8.30	− 92.7	− 95.4
sdLDL-C	− 3.7	− 30.9	− 44.4
Lp(a)	+ 2.29	− 47.2	− 40.2

*Abbreviations*: *ApoB*, apolipoprotein B; *LDL-C*, low-density lipoprotein cholesterol; *LDL-P*, low-density lipoprotein particle concentration; *Lp(a)*, lipoprotein(a); *Non-HDL-C*, non-high-density lipoprotein cholesterol; *sdLDL-C*, small, dense low-density lipoprotein cholesterol

### Phase 3 Trials of Obicetrapib

Obicetrapib is currently being studied in three pivotal phase 3 trials: the Randomized Study to Evaluate the Effect of Obicetrapib on Top of Maximum Tolerated Lipid-Modifying Therapies (BROADWAY), which has completed its enrollment of over 2500 patients with established ASCVD who require additional LDL-C-lowering [37]; the Evaluate the Effect of Obicetrapib in Patients with Heterozygous Familial Hypercholesterolemia on Top of Maximum Tolerated Lipid-Modifying Therapies (BROOKLYN) trial, which has completed enrollment of 354 participants across ten countries in North America, Europe, and Africa [36]; and the Cardiovascular Outcome Study to Evaluate the Effect of Obicetrapib in Patients with Cardiovascular Disease (PREVAIL), which is targeting enrollment of 9000 participants [38]. PREVAIL is designed to assess the potential of obicetrapib to reduce occurrences of major adverse cardiovascular events (cardiovascular death, non-fatal myocardial infarction, non-fatal stroke, and non-elective coronary revascularization) in patients with a history of ASCVD who do not have adequate LDL-C control despite being on maximally tolerated statin therapies. Results from BROADWAY and BROOKLYN are expected in 2024, whereas results from PREVAIL are expected in 2026.

### Safety and Tolerability of Obicetrapib

Obicetrapib has demonstrated excellent safety and tolerability in more than 600 patients in phase 1 and 2 clinical trials and several thousand patients enrolled in the phase 3 program to date. There have been no clinically relevant changes in vital signs, 12-lead electrocardiograms, hematology, biochemistry, or physical examinations, and single doses up to 150 mg and repeat doses of up to 25 mg were well tolerated. In phase 2 trials, most of the treatment-emergent adverse events were mild or moderate in severity, and the numbers of patients with adverse events and their severity were similar across all treatment groups, indicating no dose-dependent effects of obicetrapib treatment.

The safety concerns for CETP inhibitors sparked by torcetrapib have been largely overcome at this point; however, some scientists continue to express concern about the potential for adverse effects. One aspect of this concern relates to the extremely high plasma HDL-C levels that can be achieved with use of CETP inhibitors [68]. While low HDL-C levels are an established predictive biomarker of increased ASCVD risk, epidemiological studies have also indicated that very high HDL-C levels may be associated with increased risk of cardiovascular mortality, i.e., a “U-shaped association” [68, 69]. Nevertheless, it is difficult to interpret results from epidemiological studies where bias from uncontrolled confounders, collider stratification bias, selection bias, and reverse causation from preexisting conditions may exist. While haplotypes in the CETP gene that lead to lower CETP activity are associated with lower absolute risks of cardiovascular mortality, ischemic heart disease, myocardial infarction, peripheral artery disease, and vascular dementia, they have also been shown to be associated with a higher risk of age-related macular degeneration (AMD) [70]. A genetic association between CETP and AMD has also been reported [71] and a Mendelian randomization analysis showed a moderate hazard ratio (1.3) for variants that reduced CETP [27]. However, increased risk of AMD has never been reported in any randomized controlled trial of a CETP inhibitor, which questions the accuracy and clinical relevancy of these data [72]. Moreover, there has been interest in developing HDL as a potential therapeutic target for AMD [73].

## Proposed Mechanisms of CETP Inhibitors Alone and in Combination with Other Lipid-Altering Therapies

**Fig. 1:**
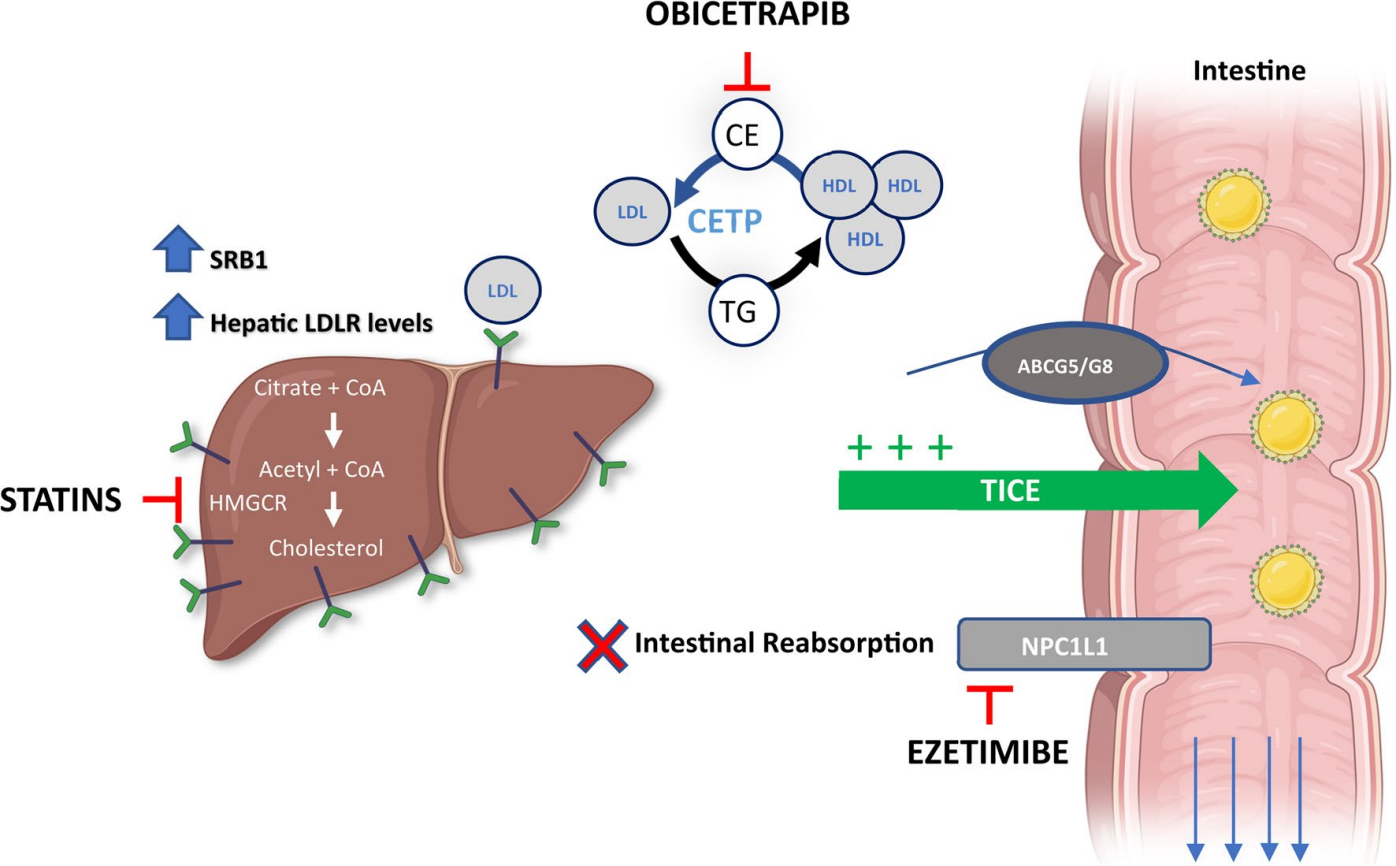
Proposed mechanisms of action for low-density lipoprotein cholesterol lowering with the combination of obicetrapib with ezetimibe on top of statins. Abbreviations: ABCG5/G8, ATP-binding cassette sub-family G member 5/member 8; CE, cholesteryl ester; CoA, coenzyme A; HDL, high-density lipoprotein; HMGCR, 3-hydroxy-3-methylglutaryl coenzyme A reductase; LDL, low-density lipoprotein; LDLR, low-density lipoprotein receptor; NPC1L1, Niemann Pick C-1 Like-1; TG, triglyceride; SRB1, scavenger receptor class B, type 1; TICE, transintestinal cholesterol excretion (used with permission from Elsevier from: Ballantyne CM, et al. J Clin Lipidol. 2023. 10.1016/j.jacl.2023.05.098, permission conveyed through Copyright Clearance Center, Inc.) [35•]

As shown in ROSE2, which combined obicetrapib with high-intensity statin and ezetimibe [35•], the extent of atherogenic lipoprotein lowering in the combination treatment group supports at least an additive effect of these three agents. This is consistent with the drugs’ different mechanisms of action as shown in Fig. 1. Obicetrapib is a CETP inhibitor, ezetimibe is a Niemann Pick C1-Like-1 inhibitor, and statins inhibit 3-hydroxy-3-methylglutaryl coenzyme A reductase. In addition to impairing the transfer of cholesteryl esters from HDL to ApoB-containing particles, CETP inhibition may also increase transintestinal cholesterol excretion, which would contribute to fecal sterol excretion, and upregulate scavenger receptor class B type 1 and hepatic LDL receptor expression [30, 74, 75]. Further, previous studies on CETP inhibitors have demonstrated that there is a substantially increased catabolic rate of LDL and ApoB, which is the primary metabolic basis for their low plasma levels. These results indicate that the LDL receptor pathway may also be upregulated by CETP inhibition [76–78]. The combination of these actions will reduce circulating LDL-C concentrations [30, 35•, 74, 75].

## Conclusions

In conclusion, obicetrapib, the next-generation, oral, once-daily, low-dose CETP inhibitor, has demonstrated excellent safety and tolerability throughout its development program and produces robust reductions in atherogenic lipoproteins when administered as monotherapy, in combination with high-intensity statins, and with high-intensity statin plus ezetimibe. As such, obicetrapib may be a promising agent for the treatment of ASCVD and it is anticipated to be the first-in-class CETP inhibitor available for clinical use.

## Data Availability

All data generated during this manuscript are included in the published article. NewAmsterdam Pharma is committed to sharing, with qualified external researchers, access to patient-level data and supporting clinical documents from eligible studies. These requests are reviewed and approved by an independent review and panel on the basis of scientific merit. All data provided are anonymized to respect the privacy of patients who have participated in the trials, in line with applicable laws and regulations.
